# Motor Overflow during Reaching in Infancy: Quantification of Limb Movement Using Inertial Motion Units

**DOI:** 10.3390/s23052653

**Published:** 2023-02-28

**Authors:** Agata Kozioł, David López Pérez, Zuzanna Laudańska, Anna Malinowska-Korczak, Karolina Babis, Oleksandra Mykhailova, Hana D’Souza, Przemysław Tomalski

**Affiliations:** 1 Neurocognitive Development Lab, Institute of Psychology, Polish Academy of Sciences, 00-378 Warsaw, Poland; 2 Graduate School for Social Research, Polish Academy of Sciences, 00-330 Warsaw, Poland; 3 Centre for Human Developmental Science, School of Psychology, Cardiff University, Cardiff CF10 3AT, UK

**Keywords:** infants, motor development, motor overflow, inertial motion units, wearables

## Abstract

Early in life, infants exhibit motor overflow, which can be defined as the generation of involuntary movements accompanying purposeful actions. We present the results of a quantitative study exploring motor overflow in 4-month-old infants. This is the first study quantifying motor overflow with high accuracy and precision provided by Inertial Motion Units. The study aimed to investigate the motor activity across the non-acting limbs during goal-directed action. To this end, we used wearable motion trackers to measure infant motor activity during a baby-gym task designed to capture overflow during reaching movements. The analysis was conducted on the subsample of participants (n = 20), who performed at least four reaches during the task. A series of Granger causality tests revealed that the activity differed depending on the non-acting limb and the type of the reaching movement. Importantly, on average, the non-acting arm preceded the activation of the acting arm. In contrast, the activity of the acting arm was followed by the activation of the legs. This may be caused by their distinct purposes in supporting postural stability and efficiency of movement execution. Finally, our findings demonstrate the utility of wearable motion trackers for precise measurement of infant movement dynamics.

## 1. Introduction

From very early on, infants undertake countless motor activities despite having limited control over their bodies. Acting on objects and moving around is one of the limited ways to learn about the environment and explore it. As motor behavior enables emerging cognitive and social skills, studying infant motor abilities is a significant part of understanding human development [1,2]. For instance, studies using a mobile (e.g., [3]) indicate that even 2-month-old infants are able to detect and memorize the contingency between their motor behavior and changes in the environment [4].

Before the age of 4 months, infants make their first attempts at reaching [5], which is one of the motor abilities crucial for interaction with the environment. This age is essential in infants’ postural development as it is the time when head stabilization becomes a priority for the infant [6], reflected in the changes of direction specificity in postural control (i.e., primary activation of the dorsal muscles during forward movements) [7]. Early on, the reaching instances rely primarily on the shoulder and elbow and are poorly executed; however, the hand and wrist eventually become more involved in the movement [5]. Nevertheless, acquiring reaching ability is much more complex than just simply possessing the skill. Research has shown high variability during the first year of life in the frequency, duration, peak, and average velocity of reaching movements [8]. Moreover, each functional movement, such as reaching, is embedded in a complex environment with particular postural, physical, or social constraints [9]. Therefore, emerging motor skills should be studied as complex, dynamic phenomena involving many components, and novel, state-of-the-art sensor technologies allow for doing so with much more detail than traditional approaches.

One of the areas of motor development that can benefit from applying novel technologies with high precision and accuracy is the phenomenon of motor overflow, i.e., the production of involuntary movements that accompany goal-directed actions [10]. Although motor overflow has been studied in detail (e.g., [4,11,12]), its exact mechanism is still debated. Possible explanations for overflow observed in infants are the lack of development of the motor system, as the nervous system is not yet entirely myelinated [13], the motor system being broadly tuned early in life, and eventually becoming more specialized [12], or symmetry being the default state of the motor system, as the two body sides are spatiotemporally linked causing simultaneous movements of body parts on both sides until the child learns how to suppress that symmetry [11]. Motor overflow has been the subject of investigation in the developmental context before (e.g., [10,11,12,14]) by comparing the performance of infants across age groups. In a study by Soska et al. [11], typically developing infants between 4.5 and 7.5 months of age performed reaching movements and out of all unimanual reaches, $\frac{4}{5}$ were accompanied by overflow movements in the non-reaching hand and legs. In another study comparing 9- and 12-month-olds, overflow in the non-acting hand and legs was more frequent for the younger group, thus showing a decrease in overflow movements with time [12]. A number of studies investigated older children and adults, and there is a general agreement that the frequency and amount of motor overflow in typically developing children decrease with age ([14,15,16,17,18]); however, its exact mechanism is still unknown. Overall, the ongoing debate on motor overflow provided more questions than answers regarding the mechanism of overflow movements. Hence, there is a need for detailed analysis of motor activity that can only be achieved with high-precision methods of recording motion.

Indeed, research on motor overflow has been mostly based on manual coding and binary classification of overflow movement (e.g., [11]). However, recent work employing motion capture methods in infancy provides new opportunities for tracking motor overflow. The most common methods of motion capture are: marker-based, i.e., they use cameras to follow markers on a body; markerless, which tracks behavior in three dimensions and detects body parts; and wearable sensors (Inertial Motion Units, IMUs) [19]. Marker-based motion capture has been used in infant studies for tracking crawling kinematics (e.g., [20]), kicking movements (e.g., [21,22]) and in the detection of abnormalities in pre-reaching behavior [23], whereas markerless motion capture was used to develop a tracking system adapted to infants [24]. Finally, the use of motion trackers is still in its early days in the study of motor development in infancy (e.g., [25,26,27,28,29]); however, they have great potential to provide unique insight in studies of motor development.

Furthermore, no studies utilizing wearable data to measure motor overflow were found. An information source that uses sensor data rather than solely manual coding is much more trustworthy, as it additionally enables quantification and inspection of the magnitude of the signal. Moreover, motion trackers enable the detection of even small changes in infants’ movements that are impossible to register with the naked eye. Therefore, studying motor activity in infancy calls for methods with high accuracy, enabling insight into infants’ spontaneous movements. In the current study, the motor behavior of infants was registered with wearable motion trackers. IMUs are a suitable research method especially in infant studies, as they can take the form of light and small sensors that can be placed on the wrists and ankles, making them comfortable but still providing excellent temporal resolution and high precision. Sensors consist of accelerometers, which can be used to investigate physical activity by monitoring changes in motor velocity, gyroscopes capturing the orientation of movement, and magnetometers registering changes in the Earth-magnetic field, enabling the recording of motor behavior in three dimensions.

In the current study, we investigated 4-month-old infants’ motor activity in the acting and non-acting limbs during spontaneous, purposeful reaching. Our main goal was to quantify the movement across the non-acting limbs during goal-directed actions and investigate the temporal distribution of motor overflow by comparing it with the motor activity of the acting limb. To this end, we first recorded infants’ limb movements using wearable motion trackers and collected detailed parameters of movement acceleration. Secondly, each reaching movement was detected and manually annotated based on the video recording. It allowed us to take a deeper look into motor behavior in the arms and legs and investigate co-activation that would traditionally be considered overflow. We expected to observe (1) differences in the temporal distribution of the motor activity between the non-acting arm and the legs and (2) diverse relationships between activation of the acting arm and the non-acting limbs depending on reaching movement.

## 2. Materials and Methods

### 2.1. Participants

In total, 47 infants and their families took part in this study. The infants—19 girls and 28 boys—were born between the 35th and 42nd week of gestation. Participants’ age ranged from 3.9 to 4.9 months (M=4.34, SD=0.29), and their birth weight was between 2360 and 4430 g. In order to be included in the final sample, participants had to be born healthy and at term, without any major genetic, metabolic, or neurological conditions and without any significant perinatal complications. None of the participants had a family history of neurodevelopmental disorders, such as autism spectrum disorder, or a family history of language disorders. All infants came from Polish-speaking, middle-class families living in a city with >1 million citizens. The study was approved by the Ethics Committee at the Institute of Psychology, Polish Academy of Sciences. All parents signed informed consent before each testing session. Quantification analysis of the reaching movements was conducted on a subsample of participants. Infants were rejected based on the lack of reaching movements (n = 15) or generating less than four of them (n = 9), due to more than 30% of missing data in any sensor (n = 2) or sensor data missing due to technical recording error (n = 1). Bimanual reaching movements were not included in the analysis as motor overflow in an arm can only be registered during unimanual reaches. The final, analyzed sample consisted of 20 infants.

### 2.2. Experimental Layout

The data in this analysis come from a longitudinal study of infant-caregiver dyadic social interactions with wearable movement sensors. The experimental task analyzed here was part of a series of tasks lasting around an hour, during which caregiver–infant dyads played different games with different sets of toys (e.g, rattles, puppets, manipulative toys, and books). These games varied in their task demands. All of them, except for the baby-gym task, which was predominantly the last one, were counterbalanced. The parent was offered to take a break between tasks to ensure the infant’s well-being. For the current analysis, we focus on movements produced during one game.

**Figure 1:**
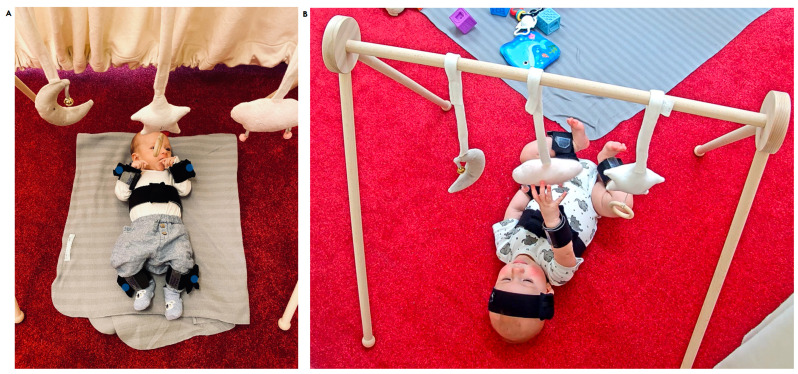
(**A**) An infant during the baby-gym task. The position of sensors on the arms and legs are marked with blue dots; (**B**) sample picture of a participant wearing motion trackers. Acquired with permission from BabyLab PAN. The signed permission of the caregiver was acquired for the publication of both images.

The experimental task involved a baby-gym, which is a useful apparatus in infant testing [30]. The task was designed as follows. A caregiver placed an infant in a supine position underneath the baby-gym (Figure 1). The baby-gym was designed to encourage purposeful reaching movements, as there were three appealing and accessible toys hanging above the participant at a reachable distance. Two of them were filled with cotton wool and had a bell attached that made a sound upon touching, whereas the third one had a string attached that, when pulled, played a lullaby melody. The strings’ lengths were adjusted to ensure that toys were within an infant’s reach. The experiment lasted for approximately 5 min unless an infant expressed visible signs of fatigue (e.g., crying or falling asleep). During this time, the caregiver was asked to fill in questionnaires and not engage in social contact with the infant; however, they remained in the same room. The purpose of the task was to elicit and record multiple, goal-directed reaching movements produced individually, without any external encouragement.

### 2.3. Equipment

The task was recorded with three remote-controlled CCTV color cameras in HD quality recording at 25 FPS, which were placed in three corners of the room. During each experimental task, an experimenter operated the camera that captured an infant best and then manipulated it during the task remotely from a separate room, which included moving the camera and zooming in and out. Movement data were recorded using 7 wearable, wireless motion trackers (MTw Awinda 3DOF, Xsens Technologies B.V., Enschede, Netherlands) predominantly at 60 Hz (except for one participant, who was measured at 40 Hz possibly due to technical malfunctioning) and an Awinda station receiver (Xsens Technologies B.V.). Each sensor was a rectangular shape (47 × 30 × 13 mm) and weighed 16 g. The dynamic accuracy was from 0.75 degrees RMS (roll/pitch) to 1.5 degrees RMS (heading) and accuracy time synchronization was ≤10 μs. The sensors were placed on the head, both arms, both legs, and two on the torso (Figure 1). The motion trackers were not restraining the movements of the parent or the infant, which was later confirmed by the caregiver. MT Manager Software (Xsens Technologies B.V.) was used to record sensor data.

### 2.4. Manual Annotation of Movement

The goal-directed reaching movements were manually annotated in ELAN 6.3 (2022) [31]. The reaching movement was defined as the period of time from the onset of the movement of an arm until the first stable, physical contact with an object, or, in unsuccessful attempts, until the end of a visible reaching try. As infants were expressing various reaching-like behaviors, four reaching categories were developed based on previous studies [32,33,34] in order to classify them more precisely (see definitions in Table 1). The movements were differentiated between being executed by the right, left, or both hands. The definition of the unimanual reaches was identical to D’Souza et al. [12]; the non-reaching hand was required to stay still or move in a targetless manner. Although bimanual movements were annotated, they were not a part of the analysis. Movement episodes in any position other than supine, as well as any clearly unintentional contact with the toy (for example, while crying), were not coded. For the purpose of synchronizing sensor and video data, for each video recording robust, isolated leg kicks were identified and annotated.

To test the reliability of reaching movement annotation, a subset of participants (n = 10, 23%) was randomly selected, and their video recordings of the baby-gym task were second-coded by another trained annotator. Inter-rater reliability was calculated in ELAN 6.3 (2022) with Cohen’s κ statistic based on Iterative Proportional Fitting (IPF) procedure used for maximum likelihood estimation, useful in analyzing cross-classified categorical data [35]. The IPF-Cohen’s κ was 0.82, which can be considered an excellent level of agreement [36].

### 2.5. Data Pre-Processing

Data from sensors were pre-processed in Python using in-house scripts incorporating pandas [37], NumPy [38], and SciPy [39] packages. The current analysis was conducted only on three-dimensional acceleration data from motion trackers. Each participant provided data from four sensors, i.e., from both arms and legs. Data from each sensor were pre-processed separately as follows. First, data from each sensor were scanned in search of one-point outliers generated most possibly due to errors in the tracking system. To this end, the absolute value of data greater than the custom threshold equal to 150 was identified. This process resulted in the identification of six one-point outliers (ranging from 39,990.56 to 75,712.38 m/s^2^) and replacing them with NaNs. The threshold of 150 greatly exceeded the inter-quartile range analysis threshold (Mdn=2.76, IQR=5.01), sometimes applied in sensor data processing [40].

Next, missing values were identified for each sensor independently, and the proportions of missing values were calculated. Data from two participants were excluded due to the proportion of missing data in at least one sensor greater than 25% (30% and 67%). The rest of the missing values did not exceed the proportion of 18% per sensor (M=0.65, SD= 2.68% of missing values per sensor), which was lower than the acceptable amount of missing data in other sensor studies [41]. The standard deviation of missing data per sensor within infants varied from 0.01 to 6.49%. Next, the missing values were interpolated using the Cubic Spline interpolation from adjacent grid points with *scipy.interpolate.interp1d* function. Then, to investigate acceleration-based data, the magnitude of acceleration for each three-dimensional acceleration data point was calculated and collapsed into one-dimensional time series [25] as follows:

(1) $$Acc=\sqrt{x{\left(t\right)}^{2}+y{\left(t\right)}^{2}+z{\left(t\right)}^{2}},$$

where *Acc* is the normalized acceleration: $x,y,z\in R^{1xN}$. Then, the data were smoothed with a 25 Hz 4th order low-pass Butterworth filter [42] using *scipy.signal.butter* following the equation:

(2) $$\left|H\left(f\right)\right|=\frac{1}{\sqrt{1+{\left(\frac{f}{f_{c}}\right)}^{2n}}},$$

where n=4 is the order, and $f_{c}=25$ Hz is a cutoff frequency [43]. Lastly, the sensor data and manual coding that were not recorded in 60 Hz were resampled to adjust to the sampling rate of other data with *scipy.interpolate.interp2d* function.

**Figure 2:**
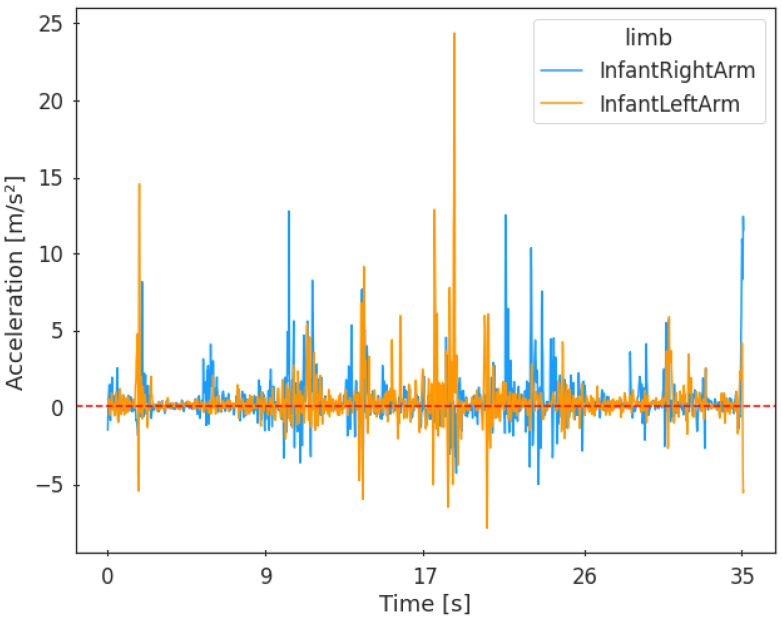
Example of the activation of the right and left infant arm during all reaching movements throughout the task.

After pre-processing, the sensor data and manual coding had to be synchronized, as sensors and cameras had to be turned on separately; consequently, there was a short delay between the data. The synchronization was performed based on the isolated activity, i.e., infant’s leg kicks, and followed the procedure from Laudańska et al. [29]. First, the acceleration data from the period of 15 s before the annotation of the first leg kick and 15 s after the annotation of the last kick were displayed on the graphical interface. Next, the period of activity peaks corresponding to kicking movements was manually identified (see Figure S1 for an exemplary activity displayed on the graphical interface). This time series of isolated acceleration peaks corresponding to leg kicks annotated based on the video recording was used to synchronize the data. To this end, the diagonal cross-recurrence quantification analysis (DCRQA) (e.g., [44]) was performed to identify the delay between manually coded infant movement and categorized sensor data with a time window that fit the data best, which was less than 3 s for the majority of data (Md=2.76, IQR=5.22). Then, the sensor data and manually coded data were plotted together, and the correctness of the data alignment was manually validated (see Figure S1 in Supplementary Materials for exemplary visualization of acceleration pre- and post-synchronization). The following analyses were conducted on the temporally aligned time series. After pre-processing, 9.8 m/s^2^ was subtracted from the acceleration data to account for gravity. Figure 2 presents an example of acceleration generated by a participant throughout the task. The code used for sensor and manual coding data pre-processing and synchronization can be found on GitHub (https://github.com/akoziol98/Processing-Sensor-Data (accessed on 23 January 2023)).

### 2.6. Data Analysis

Further analysis of the sensor data was conducted on a subsample of participants who produced at least four reaching movements (n = 20).

The analyses were conducted in Python [45] using in-house scripts. We aimed to explore differences between reaching types; therefore, all reaching movements of a certain type from all participants were collapsed and averaged. To this end, the 2-second-long period before and after a reach onset was extracted for each reaching movement and collapsed into one data frame. This window was selected based on the average duration of the reaching movement, which was t≃2 s. Finally, the activity was averaged across participants resulting in a time series of averaged acceleration. For example, for the analysis of the motor activity in the acting arm during all reaching movements produced by the infants, firstly, the process of collapsing resulted in a 240×184 data frame, where each row was one sample (60 samples constituted one second), and each column was the acceleration of the acting arm for one reaching movement. Next, the data frame was averaged across rows generating a time series of 240 samples, which constituted the motor activity of the acting arm averaged across infants.

The main goal of the analysis was to investigate the relationship between the activity generated by the goal-directed movement with the co-activation of other limbs. To this end, a series of Granger causality tests [46] were performed on pairs of time series using *grangercausalitytests* function from *statsmodels* package [47]. This function conducts four tests on the provided data; however, here we only analyze the results based on the F-test. In total, ten pairs of time series were analyzed with a Granger causality test; the acting arm paired with the non-acting arm, and the acting arm against the average of legs during all reaching instances, and then separately for four types of reaching movements (see Table 1).

The effect sizes were calculated with Cohen’s *d* [48], which has previously been applied to the analysis of time series [49]. The formula for Cohen’s *d* was as follows:

(3) $$d=\frac{M_{y}-M_{x}}{\sqrt{\frac{s_{x}^{2}+s_{y}^{2}}{2}}},$$

where $M_{x}$, $s_{x}$ are the mean and standard deviation of times series *x*.

#### Granger Causality

The Granger causality test is applied to determine whether information from one time series can provide insight into forecasting another time series. This measure is mainly applied to economic studies; however, it has also been used for human acceleration data [50] and infant–mother locomotion patterns [51]. More precisely, in the context of the current study, Granger causality can examine whether future motor activity (i.e., here acceleration) of a limb A can be predicted more accurately by including the data from the past motor activity of a limb B [51]. Hence, Granger causality is tested in the context of a lag (the maximum time period for which we move one time series and compare it with another) to check if the past values can be helpful in predicting the future information [52]. If it is, we say that limb B Granger-caused limb A, and, by extension, limb B ’leads’ limb A. It means that the movements of both limbs are related and that limb A follows the acceleration pattern of limb B [53]. For example, if during a unimanual, goal-directed motor behavior the acting arm Granger-caused the non-acting arm, it means that both of the movements are related with the acting arm being the ‘leader’ and the other arm following. Hence, it would provide an inclination that the movement of the non-acting arm was not random and was affected by the goal-directed action.

A Granger causality test is recommended to be performed under certain conditions. Firstly, each time series should be stationary [53]. Stationarity of the time series was confirmed with the Augmented Dickey–Fuller test [54] with *adfuller* function from *statsmodels* package [47]. The test was performed separately for each time series that takes part in the analysis. The second condition regarding the Granger causality test is the choice of the maximum lag [52]. Granger causality computes whether *past* values of one time series can be helpful in predicting *future* values of another one. Therefore, to perform Granger causality, we need to identify a maximum lag, i.e., the longest time period for prediction [52]. To this end, we used Akaike Information Criterion (AIC), which is a model selection criterion [55] frequently employed as a lag-selection method in Granger causality ([56]. The maximum lag was calculated separately for each pair of time series analyzed later with the Granger causality test as follows. In order to identify the maximum lag, the Vector Autoregression model, an algorithm for prediction between time series [57], was applied to a pair of time series and fit all possible lags (i.e., 120). Then, the AIC was calculated for each model and the lag for which the model had the lowest value of AIC was selected. The best lag was equal for all pairs of time series, which was 1 sample ($\frac{1}{60}$ of a second).

## 3. Results

### 3.1. Reaching Descriptives

A sample of 20 infants during the baby-gym task made a total of 297 reaches (M=14.85, SD=11.60). Most of the reaching movements were produced only by one hand (n = 184) or by the left (n = 93) or the right (n = 91) one. The performance in terms of types of reaching movements (see Table 1) was varied. A substantial amount of reaches ended with a grasp (n = 89), some with a touch (n = 61). The reaching movements were predominantly successful, i.e., ended with physical contact. Unsuccessful reaching attempts were observed less often (n = 34) and in hardly any participants was their reaching performance composed of more than 50% of unsuccessful reaches. No relationship was found between the reaching performance and age. The age did not correlate with either the overall number of the reaching movements (ρ=0.02, p=0.93) or the number of unsuccessful reaches (ρ=−0.14, p=0.56). Therefore, although participants varied in their level of reaching skill, these differences are not simply the result of their age.

### 3.2. Overflow Quantification

The findings of the relationship between the acting arm and the non-acting arm are summarized in Table 2, whereas the acting arm and the legs are in Table 3. A series of Granger causality tests were performed on pairs of time series in order to estimate their relationship. Each time series contained acceleration from the time period of two seconds before and after particular reaching instances averaged across those reaching movements. Hence, each time series consisted of 240 samples.

**Figure 3:**
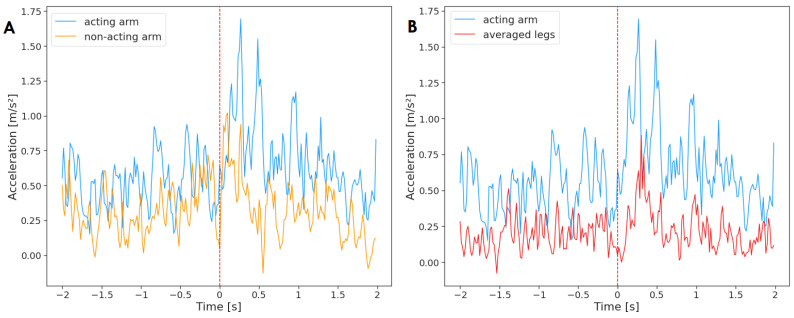
Acceleration of the acting hand with (**A**) the non-acting arm and (**B**) legs mean, averaged across all reaching movements (n = 184). The plot displays activity two seconds before and two seconds after the onset of the reaching movement (marked by a red, dashed line at time = 0). The standard deviation has not been added to the plot to improve clarity.

First, all reaching movements were analyzed. To this end, a time series containing acceleration of the acting arm averaged across all unimanual reaching movements (n = 184) was tested against an analogous times series of the non-acting arm (Figure 3). The results of the Granger causality test indicate that the non-acting arm Granger-caused the movement of the acting arm (F(236)=6.88, p=0.01, d=1.22). There was no reverse effect (F(236)=0.65, p=0.42, d=1.22). It means that the motor activity of both arms was related, and the information from past values of the non-acting arm was helpful in predicting future values of the acting arm. In other words, the non-acting arm was the ‘leader’ that the acting arm followed.

**Figure 4:**
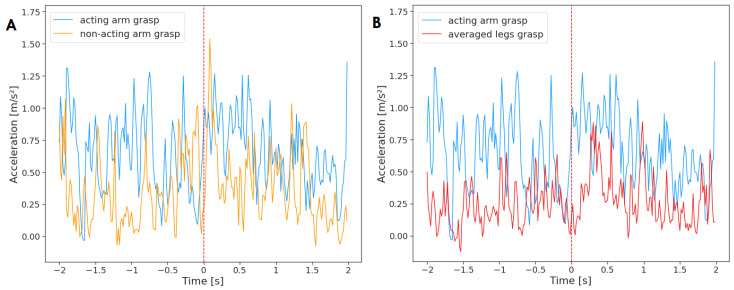
Acceleration of the acting hand with (**A**) the non-acting arm and (**B**) legs mean, averaged across reaching movements that ended with a grasp (n = 89). The plot displays activity two seconds before and two seconds after the onset of the reaching movement (marked by red, dashed line at time = 0). The standard deviation has not been added to the plot to improve clarity.

Next, a Granger causality test was performed between the acting arm and the averaged acceleration of legs for all unimanual reaching movements (n = 184) (Figure 4). The results indicate that the acting arm Granger-caused the activity of legs (F(236)=19.57, p<0.001, d=1.93); however, the effect was not reciprocated (F(236)=0.08, p=0.78, d=1.93). Therefore, a relationship between the motor activity of the acting arm and the legs was found, and the acceleration of the acting arm provided insight into forecasting the motor activity of the legs.

Next, the reaching instances were analyzed within particular reaching types. To this end, the acceleration of the acting and the non-acting arm and legs during only a certain type of reaching movement was collapsed and averaged.

The activity of limbs for reaches that ended with a grasp (n = 89) can be found in Figure 4. The Granger causality test did not find any significant relationship between the limbs. The activity of the non-acting arm did not Granger-cause the acting arm (F(236)=2.03, p=0.16, d=0.97) and vice versa (F(236)=0.08, p=0.78, d=0.97). The activity of the legs did not Granger-cause the activity of the acting arm (F(236)=0.46, p=0.50, d=1.56) and the same for the opposite test (F(236)=1.88, p=0.17, d=1.56).

**Figure 5:**
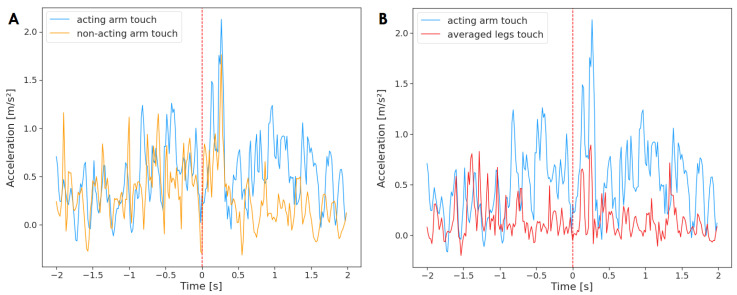
Acceleration of the acting hand with (**A**) the non-acting arm and (**B**) legs mean, averaged across reaching movements that ended with a touch (n = 61). The plot displays activity two seconds before and two seconds after the reach onset (marked by red, dashed line at time = 0). The standard deviation has not been added to the plot to improve clarity.

Figure 5 depicts the averaged activation of the acting arm and the non-acting limbs for reaching movements that only ended with a touch (n = 61). For this reaching type, the acting arm Granger-caused neither the non-acting one (F(236)=0.74, p=0.39, p=0.64), nor the legs (F(236)=0.30, p=0.58, d=1.30). The effect was also not significant for the non-acting arm (F(236)=1.54, p=0.22, d=0.64) and the legs (F(236)=1.73, p=0.19,1.30) on the acting arm.

**Figure 6:**
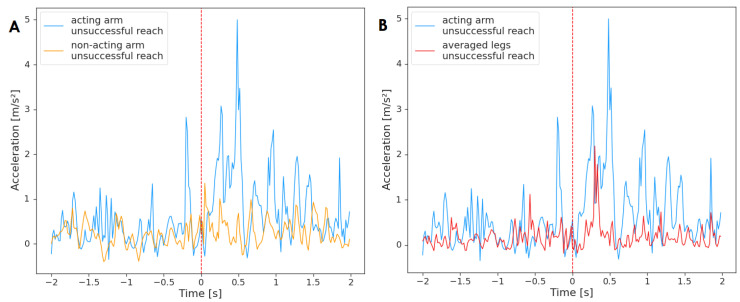
Acceleration of the acting hand with (**A**) the non-acting arm and (**B**) legs mean, averaged across unsuccessful reaching movements (n = 34). The plot displays activity two seconds before and two seconds after the onset of the reaching movement (marked by red, dashed line at time = 0). The standard deviation has not been added to the plot to improve clarity.

Finally, Figure 6 depicts averaged acceleration of the limbs for unsuccessful reaching attempts (n = 34). The non-acting arm did not Granger-cause the acting arm during unsuccessful reaching movements (F(236)=2.02, p=0.16, d=0.70) and similarly for the opposite test (F(236)=0.24, p=0.63, d=0.70). No relationship was registered between the activity of the arms; however, the legs and the acting hand were found to be related. The legs did not Granger-cause the activity of the acting arm during unsuccessful reaching movements (F(236)=0.14, p=0.71, d=0.82), but the acting arm Granger-caused the average of legs (F(236)=7.74, p=0.01, d=0.82). Therefore, the acting arm was the ‘leader’, and its past activity was helpful in predicting the acceleration of legs in the future.

## 4. Discussion

The main purpose of this research was to study the distribution of motor activation across limbs during purposeful reaches and investigate whether the acceleration of the acting arm is related to the patterns of activation in the non-acting limbs.

The reaching abilities varied substantially among participants, which might be expected in 4-month-olds who are in the transition period toward the acquisition of reaching. Half of the infants in the total sample made less than four reaches and over 30% of the sample did not make a single one, which suggests unimanual reaching was still a novel motor skill for some infants, requiring further practice and refinement.

When all reaching movements were considered, the activity of the acting arm was found to be related to the other arm and the legs, which indicates that the movement of the non-acting arm was not random and dependent on the goal-directed action. However, differences in the temporal distribution were registered, as the non-acting arm was ’leading’ the acting arm, whereas the acting arm was a ‘leader’ to the activity of the legs. Therefore, it can be concluded that the activation in the non-acting arm was on average before the goal-directed movement, whereas the legs predominantly activated after the onset of the reaching movement. This suggests that the movements of the non-acting limbs could have a functional explanation in the context of a developing motor system. The temporal sequence of activation of the subsequent body parts could be related to the maximization of reaching performance, supported by findings on the relationship between postural control and reaching movements in new reachers [58]. Reaching in the supine position is a challenge for the infants as they need to deliver additional force than, e.g., in the vertical position, to overcome gravity [59]. Hence, the leg activity following the reaching movement could provide additional postural control, allowing for a more stable execution of the reaching movement [9]. Similarly, activation of the non-reaching arm before the onset of the reaching movement could be related to the stabilization of posture [60]. Therefore, due to the diverse patterns of activation, there is a possibility that the motor activity recorded in the non-acting limbs during goal-directed reaching movements could, in fact, be instances of purposeful movement related to postural control. Further research on the co-activation of limbs with a deeper exploration of posture stabilization is required.

Further analysis across the reaching types provided additional insight into the motor activity of the non-acting limbs. Even though there was a relationship between the activation of limbs for all reaching movements, hardly any relationship was found for particular reaching types, except for unsuccessful reaching movements, during which the acting arm was ’leading’ the legs. The difference in temporal relationship underlines differences in those reaching types, and the activation of the non-acting limbs could again illustrate the diverse patterns of postural adjustments that had to be made while executing each reaching movement. However, it is possible that the amount of the reaching movements of certain types was insufficient to test the relationship.

Furthermore, investigating motor activity in new reachers provides valuable insight into how this skill is developed, which was reflected here in the multiplicity of the reaching types. In previous studies (e.g., [11]), the analyzed reaching movements were reduced to only grasps, whereas the wide range of movement types observed here suggests that a more nuanced approach is needed [9]. The current results indicate that the motor activity of the non-acting limbs varies across limbs and types of reaching movement. Therefore, the results provide support for identifying major distinctions within the co-activation of limbs depending on the execution of the goal-directed behavior, and in which limbs the extraneous movement was registered. Moreover, inconsistency in the issue of which limb was the ‘leader’ and which was the ’follower’ provides an inclination that the movement of the non-acting limbs could have a purpose, possibly relating to postural control.

### 4.1. Sensors’ Usability

Given the portability, mobility, low hardware cost, small size, and low weight, wearable IMU technology has tremendous potential in infant studies in both laboratory and natural environments. First, it enables conducting day-long and continuous recordings of spontaneous movements of infants [27]. Second, multiple and flexible measurements can be obtained at a wider age range. This is especially important for accurately describing typical, daily infant activity, where the most reliable option is to collect data across consecutive days [61]. Third, wearables facilitate the simultaneous data acquisition from two or more people [62], which is key for studying how social interactions with caregivers influence movement patterns in infants and how dyadic coordination emerges. Finally, as devices become easier to use and data transfer techniques improve, parents might be even trained to acquire infants’ data in their natural environment without the constant presence of an experimenter (see example in [63,64]).

In our study, the majority of participants did not display any inconvenience related to wearing sensors. After the experimental session, the caregivers were asked whether they observed any differences in the infant’s motor behavior as a result of wearing IMUs. Only two parents from the final sample reported changes in behavior—for the great majority of infants, the sensors did not affect their performance. Furthermore, the recording of reaching actions was the last task in a session of several interaction games with caregivers, so infants had around 40–60 min of previous experience with wearing equipment to get used to it. Experimenters had a live view of the infant’s performance during the session through CCTV cameras and corrected the placement of sensors between tasks if they were loose or displaced. They were also making sure that the sensors were put in the same place on each limb and that the Velcro bands that held the sensors on the wrists and ankles were tightly adjusted. In addition, the caregivers were instructed not to interfere with the sensors’ placement and instead to inform the experimenter if any sensor placement corrections were necessary. In addition, the data used in the present paper were based on manual annotation of reaching movements and the coders only marked episodes when the sensors were placed correctly. Finally, in our previous validation paper [65], we showed that the computer vision algorithm (DeepLabCut [66]) and wearable sensors provide comparable quantitative data on spontaneous limb movements in infants at the age of 4–5 months. Therefore, data acquired with IMUs are a precise source of information regarding the motor activity, and additionally, they are a flexible method that does not require computationally expensive video processing.

### 4.2. Limitations and Further Directions

Participants before performing the baby-gym task took part in a series of other tasks lasting around 40–60 min. Consequently, there is a possibility that the order of the task influenced the participants’ performance or that the infants were too tired to contribute to the current task fruitfully. On the other hand, the baby-gym task was the only one that the infant was performing alone (without being in interaction with the parent); therefore, it was required that the infant felt most comfortable in the new place. Secondly, the manual coding and the sensor data had to be synchronized, which was carried out based on the isolated activity (i.e., leg kicks). Consequently, the synchronization process could potentially cause inaccuracy in data alignment; however, the procedure applied in the current project was found to be useful in other research [29]. Finally, there was a limited amount of particular types of reaching movements, which possibly affected the analysis. Therefore, in future studies, the experimental procedure should be adjusted to prompt a higher quantity of each reaching type.

Findings from the current study provide opportunities for further research. Most importantly, the development of motor skills over time can be explored by comparing the data obtained at the age of 4 months with later time points. Additionally, it might be investigated to see if the co-activation of limbs at 4 months predicts the development of fine and gross motor skills at later stages. Finally, exploration of activity in the non-acting limbs during reaching at 6 and 9 months can help to establish a developmental trajectory of motor overflow.

Wearable sensor systems in general can be applied to the clinical field as a health monitoring tool in infancy (see a review of sensor systems [67]). Moreover, sensors can also contribute to the early diagnosis of motor dysfunctions, as they can detect subtle motor impairments that are a precursor to clinically observable motor disorders [68]. For instance, differences in activation patterns were found between typically developing infants and infants at risk for developmental disorders when day-long leg movements were recorded with wearable sensors [69]. In addition, Machine Learning models can be applied to sensor data and be useful in identifying abnormalities in coordination patterns in at-risk infants [70]. Consequently, early markers of neurodevelopmental disorders could be recognized at the level of an individual. Given the usability of wearable sensors, they can also enable regular monitoring of motor activity of infants at risk for developmental delay at home, and additionally measure the effectiveness of early interventions [64,71]. Finally, our data on infants’ spontaneous reaching movements can be useful in registering early motor abnormalities. For instance, longitudinal monitoring of reaching-to-grasp movements of at-risk infants starting from 6 months revealed that infants later diagnosed with autism spectrum disorder had significantly worse reaching performance [72]. Since reaching is one of the earliest complex motor skills that infants need to master, monitoring its development with wearable motion trackers in infants that are at an elevated likelihood of developmental difficulties may contribute to improvements in an early clinical diagnosis.

## 5. Conclusions

This is the first study investigating motor overflow with wearable motion trackers. In conclusion, in the current study, we used wearable motion trackers to measure motor activity across limbs during goal-directed reaching movements of 4-month-old infants. The findings from a series of Granger causality tests conducted between the acting arm and the non-acting arm and legs indicate diversified patterns of activation under certain conditions. When all reaching movements were analyzed, the activity of the acting arm and the non-acting limbs were related; however, the movement of the non-acting arm was followed by the acting arm, whereas the relationship was the opposite for the activation of the legs. The temporal differences in activation could be explained by diverse needs for postural adjustment to maintain body stability, indicating the purpose behind the movement patterns of the non-acting limbs. The effects were predominantly insignificant when only particular types of reaching movements were considered. The results support further analysis of the co-activation of limbs, as the movement patterns had major differences depending on the type of purposeful reaching and the non-acting limb.

## Figures and Tables

**Table 1:** Definitions of the reaching movements present in the analysis.

Movement Name	Definition
Grasp	An infant’s hand moves toward the toy, touches it and tightens the hand around it, while the remaining limbs are not involved in any other purposeful action.
Touch	An infant’s hand moves toward the toy, touches it and lets go without any further contact.
Unsuccessful reach	An infant moves the hand toward the toy and misses it but arrests the hand in midair, with the arm stretched out and gaze focused on the toy in a visible attempt to try to touch the object.

**Table 2:** Results of the bilateral Granger causality test between the acting arm and the non-acting arm.

Non-Acting Arm Leading the Acting Arm			
**Reaching Type**	**No. of Reaches**	**F-Statistic**	**Cohen’s** ***d***
All	184	6.88 *	1.22
Grasp	89	2.03	0.97
Touch	61	1.54	0.64
Unsuccessful reach	34	2.02	0.70
**Acting Arm Leading the Non-Acting Arm**			
**Reaching Type**	**No. of Reaches**	**F-Statistic**	**Cohen’s** ***d***
All	184	0.65	1.22
Grasp	89	0.08	0.97
Touch	61	0.74	0.64
Unsuccessful reach	34	0.24	0.70

Note: * *p* < 0.05. Significant results highlighted in gray.

**Table 3:** Results of the bilateral Granger causality test between the acting arm and the legs.

Legs Leading the Acting Arm			
**Reaching Type**	**No. of Reaches**	**F-Statistic**	**Cohen’s** ***d***
All	184	0.08	1.93
Grasp	89	0.46	1.56
Touch	61	1.73	1.30
Unsuccessful	34	0.14	0.82
**Acting Arm Leading the Legs**			
**Reaching Type**	**No. of Reaches**	**F-Statistic**	**Cohen’s *d***
All	184	19.57 ***	1.93
Grasp	89	1.88	1.56
Touch	61	0.30	1.30
Unsuccessful	34	7.74 *	0.82

Note: * *p* < 0.05, *** *p* < 0.001. Significant results highlighted in gray.

## Data Availability

The datasets presented in this article will be available upon request from the corresponding authors following an embargo period from the date of publication to allow for the finalization of the ongoing longitudinal project. The computer code used in this study is openly available in GitHub: https://github.com/akoziol98/Processing-Sensor-Data (accessed on 23 January 2023). Requests to access the datasets should be directed to ptomalski@psych.pan.pl.

## Supplementary Materials

The following supporting information can be downloaded at: https://www.mdpi.com/article/10.3390/s23052653/s1, Figure S1: Example of the activation pre- (top) and post-synchronization (down).

## Abbreviations

The following abbreviations are used in this manuscript:

IMUs	Inertial Motion Units
IPF	Iterative Proportional Fitting
DCRQA	Diagonal Cross-Recurrence Quantification Analysis
AIC	Akaike Information Criterion
IQR	Interquartile Range
M	Mean
Md	Median
SD	Standard Deviation
