# Supplementary figures and images for: Motor Overflow during Reaching in Infancy: Quantification of Limb Movement Using Inertial Motion Units
Source: Sensors (Basel). 2023 Feb 28;23(5):2653. doi: 10.3390/s23052653 (PMC10007533; doi:10.3390/s23052653)

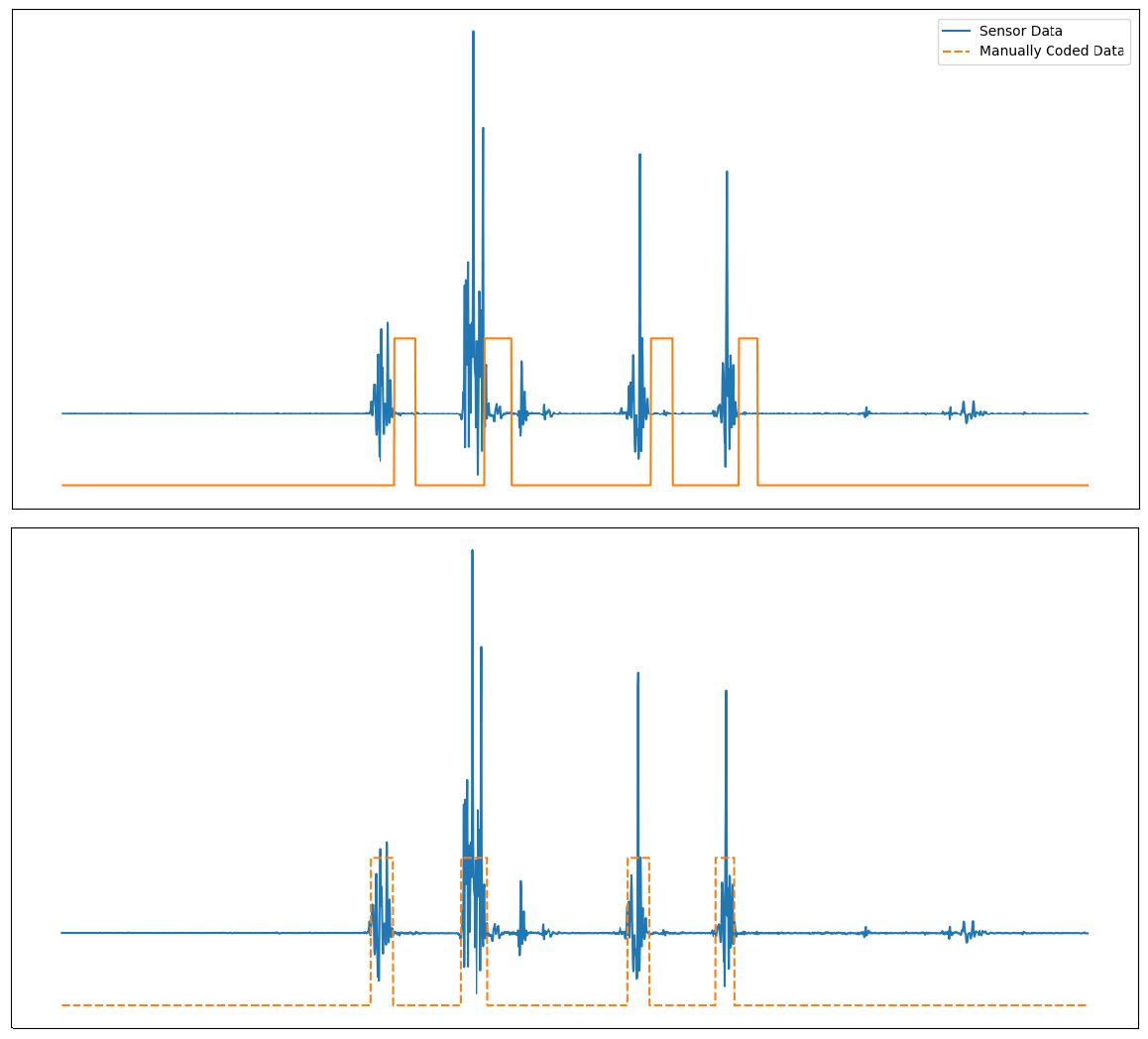

Supplement: Supplementary file 1 [file sensors-23-02653-s001.zip › synchronization.png]
